# An Address upon Chayes Movable-Removable Bridge-Work

**Published:** 1921-04

**Authors:** Walter G. Hine


### AN ADDRESS UPON CHAYES MOVABLE-REMOVABLE BRIDGE-WORK

BY WALTER G. HINE, D.D.S.

I esteem it a great pleasure to address you at this time upon a subject so interesting and of such vital importance to the profession.

The Chayes system of movable-removable bridge-work is a system with which some of you are more or less familiar, some more because of sincerity of purpose they have worked hard with open minds to receive; others less, because their minds are already filled with ideas and closed to others they do not see.

Some years ago I started to practice the Chayes system with only a slight comprehension of the principles involved; in my own mind, however, I knew *all* about it and freely discussed its merits and deficiencies. Later I became resident director of the Chayes Clinic in New York City, where I enjoyed every minute of the many hours I have had with Dr. Chayes himself and where I have had an excellent opportunity to study the system from the time of its conception up to date, to go through the scrap-heap and see the different meters, the various forms of attachments

(some of which have been reconceived or copied and are manufactured today and an attempt is made to use them); all have failed to meet the requirements exacted from them by the Chayes system, and today I find the criticisms I offered were made through a lack of knowledge of the workings of the system and it was impossible for me to add to or detract from this wonderful conception of Dr. Chayes, executed by his corps of skilled mechanics and presented to us.

Gentlemen, with your kind indulgence, I will read three pages from the regular teaching outline. I have selected these three pages because they treat of the saddle alone—that you may see the requirements a saddle has to meet if it is to become a part of the Chayes appliance, destined to become a part of the human apparatus, remembering that this human apparatus is a dynamic institution—not static. Parts of the anatomy are constantly changing in form, position and sometimes composition. You will notice that the Chayes worker does not care to be the one who is to inhibit or interfere with these changes, but he is the operator who says to himself: "My apparatus is the only thing in this being that is not living. I don't want it to augment or retard any process or processes that are perpetuating life in this body. On the contrary, I want it to work with them."

SADDLES

The saddle is that part of a bridge which rests upon the mucosa and supports the artificial tooth or teeth. The greater area covered by the saddle, the more stable will be the support of the bridge under the stress of mastication. The size and design of saddles must vary as the kind and number of artificial teeth supplied and the conditions of the underlying tissue. Single anterior teeth may be supplied with a saddle no larger than the gingival circumference of the teeth themselves, providing the artificial tooth is attached to parallel natural abutments by means of parallel attachments at its mesial and distal extremity. The saddle as a means of relieving stress upon abutments is a far more important factor in the upper mouth to be fitted with posterior bridges, than in the lower, because the mandible is the moving member of the masticating machine, or the power arm. The ultimate shape of a saddle must be calculated not to encroach upon the gingival circumference of the teeth adjacent to the edentulous space. A saddle should not be movable upon the mucous membrane, but should move with it. It may be correctly compared to a pontoon bridge moving with the tidal motion of the liquid upon which it rests. The area of the saddle should equal the area of the roots of those teeth which are being replaced with the artificial ones carried on the saddle. A saddle must be perfectly adapted to the subadjacent tissue. A properly cast saddle is more perfectly adapted to the subadjacent tissue than one which is swaged. The saddle should be as thin as the stress to which it will be subjected will permit, and it should never be a bulk that will prove obtrusive to any of the surrounding and adjacent or subadjacent tissue.

### PHYSIOLOGICAL FUNCTIONS OF A SADDLE

The muscles of mastication are the motor, the artificial teeth resting upon the saddle are the driving or vibrating shaft, and the saddle is the applicator which massages the subadjacent mucosa and underlying structures when the artificial teeth are in function. The more perfect the occlusion of the artificial teeth, the more rhythmic the periodicity of the intermittent pressure transmitted through the saddle intermediary to the subadjacent tissues. The more rhythmic the periodicity of the intermittent pressure, the more rhythmic the waves of vibration engendered by the intermittent pressure and transmitted through the saddle applicator, thence to the adjadcent and subadjacent living tissues, and the most perfect will be the vaso-motor action of the nutrient vessels of these tissues. All the foregoing is in harmony with the statement that a bridge is an artificial partial denture resting upon a floating base, its motion being aided to a degree limited by parallel attachments constructed in accordance with the geometric conception of the arc being tangent to a straight line. Again, teeth, natural teeth, our own teeth, move in the exercise of their function; they move in various directions, the degree and direction varying as the complexity of function, being little, and more or less simple for the incisors, and intense and multi-directional for trituration.

### EVILS OF IMPROPERLY CONSTRUCTED SADDLES

An imperfectly adapted saddle, which can not move while the teeth are in function, will always cause resorption. A saddle which snaps into position, then becomes as stable as a gate on loose hinges, will always cause resorption. A saddle, though perfectly adapted to the mucosa, but held there against all lateral displacement, will always cause hyperemia and finally resorption. A saddle, though perfectly adapted to the underlying mucosa, but held there against lateral vertical displacement, will bring about a distinct pathological change, and finally resorption of adjacent and subadjacent structures.

### CORRECT CONSTRUCTION OF SADDLES

A saddle must be perfectly adapted to the underlying mucosa and held in place by parallel attachments which engage parallel abutments, and which will allow a limited vertical, bucco-lingual, lingo-buccal and to a very limited degree a mesio-disto yield. The extent of the vertical yield is determined by the elastic limit of the subadjacent tissue upon which the saddles rests; a halt must be called before the limit of displacement has been reached. The extent of the bucco-lingual and lingo-buccal yield is controlled by the degrees of the arc created upon the attachments and the degree of the arc is determined by the condition of the subadjacent tissue, being greater for the soft and easily displaced tissue, and less for hard and unwillingly yielding tissue. The mesio-distal yield is effected by having the attachments which hold the saddle in position consist of double metallic plates, elastic in action, with a minute space between them. Impression for saddles in the lower mouth should be muscle trimmed.

Pathology has its inception when a fixed bridge is set or cemented into position; the amount of catabolism being governed by the seriousness of the handicap imposed upon the human economy. Again, mechanical interference with a normal circulatory exchange at the gingivus, either by creating too much tension on the free margin or allowing an utter collapse. Or interference with the secretory process of the mucous glands. Or depriving a tooth in any way of its right to vibrate and its supporting structures and increasing the molecular activity, to enhance their specialized powers to extract the things that preserve their metabolic balance. By allowing a saddle to exert a non-intermittent, or, in other words, a constant pressure on the mucous membrane, and so on.

The clearer the conception of the various causes of pathology, the better fitted is the operator to avoid pathology or maintain physiological conditions; isn't that true?

Should we not then encourage the professional man in whose care people are placing their human bodies for repair, for adopting a system or procedure, if you please, that conforms so closely to life principles, for executing work, which built and placed, expresses absolute harmony with the oral apparatus and the processes of life?

In the first place, the system requires no unnecessary pulp removals.

So anxious is the author or inventor to retain the vitality of healthy teeth, that he has devised and caused to be manufactured five different sizes of bucco-lingual attachments for use in vital teeth, that a maximum efficiency for the amount of tooth structure removed may be obtained and yet house the member within the original circumference of the tooth, lest some force or stress may be exerted, foreign to that which it was accustomed to.

In addition to the bucco-lingual attachments which are generally used wherever possible, we have the upright and split-pin and tube (enlarged fac-similes were displayed) which may be used in pulpless teeth and root ends.—*Pacific Dental Gazette.*

